# Acknowledgment to Reviewers of *Journal of Intelligence* in 2020

**DOI:** 10.3390/jintelligence9010006

**Published:** 2021-02-01

**Authors:** Journal of Intelligence Editorial Office

**Affiliations:** MDPI AG, St. Alban-Anlage 66, 4052 Basel, Switzerland

Peer review is the driving force of journal development, and reviewers are gatekeepers who ensure that *Journal of Intelligence* maintains its standards for the high quality of its published papers. Thanks to the cooperation of our reviewers, in 2020, the median time to first decision was 33 days and the median time to publication was 97.5 days. The editors would like to express their sincere gratitude to the following reviewers for their precious time and dedication, regardless of whether the papers were finally published:
Acar, SelcukLange, JensAckerman, Phillip L.Lautrey, JacquesAgnoli, SergioLe Boutillier, NicholasAmbrose, DonLecerf, ThierryAustin, ElizabethLechner, ClemensAxelrod, MichaelLeikin, MarkBeath, AlissaLeikin, RozaBeckmann, Jens F.Lewis, MichaelBirney, DamianLignier, BaptisteBobak, Anna K.Lim, Kenneth Y. T.Boyle, Gregory J.Lubart, ToddBruckmaier, GeorgLwi, Sandy J.Buczylowska, DorotaMacLean, RoryCaffò, AlessandroMayer, JohnChen, YunxiaoMcShane, BlakeleyChristensen, AlexanderMehu, MarcCobo, María José GutiérrezMills, CaitlinColom, RobertoMiroshnik, KirillConway, AndrewMorningstar, MicheleCropley, DavidMurphy, Kevin R.D’Amico, SimonettaMurphy, RaeganDauvier, BrunoNador, JeffDavis, SarahNeubauer, AljoschaDe Ribaupierre, AnikNolte, MarianneDebatin, TobiasOlderbak, SallyDemetriou, AndreasOvington, LindaDenervaud, SolangePartchev, IvailoDoebler, PhilippPenke, LarsEdelsbrunner, PeterPérez Marfil, Maria NievesEidels, AmiPietschnig, JakobElfenbein, Hillary AngerPohl, SteffiEsteban-Navarro, Miguel-ÁngelPosner, MichaelFerrandiz, CarmenPrieto, Mercedes FerrandoFischer, AndreasRecio, GuillermoFontaine, JohnnyRedifer, JenniForthmann, BorisReid, ScottFrischkorn, Gidon T.Renta-Davids, Ana-InésGignac, GillesRindermann, HeinerGoh, Jin X.Robitzsch, AlexanderGoldhammer, FrankRooij, Alwin DeGolino, HudsonRunco, Mark A.Goring, Sara AnneRuss, SandraGottfredson, LindaSavi, Alexander O.Grabner, Roland H.Schermer, Julie AitkenGredlein, Jeffrey M.Schlegel, KatjaGrigoriev, AndreiSchmitz, FlorianHall, Judith A.Schroeders, UlrichHalpern, DianeSchubert, Anna-LenaHannon, BrendaSchulze, JulianHarris, AlexandraSchweizer, KarlHass, Richard W.Sergio, MorraHildebrandt, AndreaShcherbakova, Olga V.Hilger, KirstenShearer, BrantonHo, Moon-HoSilvia, PaulHommel, BernhardSimonet, DanielHuber, LudwigSimonton, Dean KeithHülür, GizemSkalicky, StephenIorga, MagdalenaStemmler, MarkJauk, EmanuelSternberg, Robert J.Joseph, DanaStrang, CarolineJung, RexTijmstra, JesperKafetsios, KonstantinosVonk, JenniferKan, Kees JanWai, JonathanKarpinski, Ruth I.Weirich, SebastianKarwowski, MaciejWieckowski, Andrea TrubanovaKenett, Yoed N.Wilhelm, OliverKievit, RogierWilson, RachelKleka, PawełXu, GongjunKovacs, KristofZabelina, Darya L.Kretzschmar, AndréZafeiris, AnnaKroesbergen, Evelyn H.Zehner, FabianKuo, Susan S.Zeidner, MosheKurihara, SatoshiZiccardi, StefanoKurvers, RalfZiegler, Matthias

